# The Latent Structure of Child and Adolescent Psychopathology and its Association with Different Forms of Trauma and Suicidality and Self-Harm: LATENT STRUCTURE OF PSYCHOPATHOLOGY

**DOI:** 10.1007/s10802-022-00925-1

**Published:** 2022-04-27

**Authors:** Philip Hyland, Thanos Karatzias, Julian D. Ford, Robert Fox, Joseph Spinazzola

**Affiliations:** 1 Department of Psychology, Maynooth University, Kildare, Ireland; 2 Department of Psychology, Edinburgh Napier University, Edinburgh, Scotland; 3 Department of Psychiatry, University of Connecticut School of Medicine, Farmington, CT USA; 4 School of Business, National College of Ireland, Dublin, Ireland; 5 The Foundation Trust, Melrose, MA USA

**Keywords:** HiTOP, Psychopathology, Child and adolescent, Trauma, Suicidality, Self-harm

## Abstract

The Hierarchical Taxonomy of Psychopathology (HiTOP) is a multidimensional and hierarchical model of the latent structure of psychopathology. While HiTOP has received much support in child/adolescent community samples, fewer studies have assessed this model in clinical samples of children/adolescents. Here, we modelled the latent structure of 45 symptoms of psychopathology from a clinical sample of children/adolescents and assessed how dimensions of psychopathology were related to specific forms of trauma and suicidality/self-harm. Clinician-derived assessments were obtained from 507 people aged 7–18 years. Confirmatory factor analysis was used to determine the optimal fitting model, and structural equation modelling was used to estimate associations with trauma exposure and suicidality/self-harm. The best fitting model(s) included five first-order factors reflecting Fear, Distress, Externalizing, Thought Disorder, and Traumatic Stress, with a higher-order general factor (*p*) accounting for the covariation between these factors. Unique associations were identified between specific forms of trauma and each dimension of psychopathology. *p* was strongly associated with suicidality/self-harm, and of the first-order factors, Distress was most strongly associated with suicidality/self-harm. Findings support the predictions of HiTOP that the latent structure of child/adolescent psychopathology can be effectively described by a multidimensional and hierarchal model. Moreover, we found tentative evidence for a unique dimension of Traumatic Stress psychopathology. Our findings also highlight the unique associations between specific forms of early life trauma and specific dimensions of psychopathology, and the importance of Distress related psychopathology for suicidality/self-harm in children and adolescents.

## Introduction

There are disagreements among psychological scientists about how best to conceptualise psychopathology. For the last century the ‘neo-Kraepelinian’ view has dominated psychiatry and clinical psychology positing that psychopathology arises due to the occurrence of discrete psychiatric disorders; that these disorders can be defined and identified by specific symptoms; and that these disorders are caused by biological pathology (Engstrom & Kendler, 2015). However, evidence of diagnostic comorbidity, disorder heterogeneity, poor diagnostic reliability, and non-specificity in symptoms, risk-factors, neurological functioning, and psychopharmacological and psychotherapeutic treatment effects has led many researchers and clinicians to explore transdiagnostic, dimensional frameworks for understanding the structure of psychopathology (Caspi & Moffitt, 2018; Conway et al., 2019; Fusar-Poli et al., 2019; Kotov et al., 2021; Krueger et al., 1998; Michelini et al., 2021; Ringwald et al., 2021).

One prominent model is the Hierarchical Taxonomy of Psychopathology (HiTOP) (Kotov et al., 2017; DeYong et al., 2020). HiTOP is a hierarchical and multidimensional model of the structure of psychopathology. At the lowest level of the model are the panoply of distressing symptoms (e.g., worry, low mood, anhedonia). Recognizing that symptoms co-occur in predictable ways, the next level of the hierarchy includes ‘syndromes’ (e.g., depression, generalized anxiety, substance misuse, paranoia). HiTOP assumes that these syndromes covary and such covariation is explained by several higher-order latent factors termed ‘subfactors’ (e.g., Fear, Distress, Mania). Multiple subfactors exist which themselves covary due to a small set of higher-order dimensions termed ‘spectra’ (e.g., ‘Internalizing’, ‘Though Disorder’, ‘Externalizing’). These spectra also covary and a superordinate general factor of psychopathology – termed the ‘*p*’ factor (Caspi & Moffitt, 2018) – is posited to account for these covariations.

The development of HiTOP was informed by dimensional-based research in child and adolescent psychopathology (Achenbach & Edelbrock, 1981). HiTOP makes no distinction between the structure of psychopathology in adults versus young people and there is considerable evidence that the structure of psychopathology in young people is consistent with the HiTOP model. Patalay et al. (2015) examined the latent structure of a broad set of indicators of psychological distress in a community sample of children aged 11–13 years (*N* = 23,477) and found evidence of two broad dimensions of psychopathology (Internalizing and Externalizing), along with the *p* factor. Subsequent studies with a diverse range of child and adolescent samples have reported similar results, including evidence of subfactors under the Internalizing and Externalizing spectra (Afzali et al., 2018; Carragher et al., 2016; He & Li, 2021; Laceulle et al., 2015; Michelini et al., 2019).

Two notable gaps exist in this literature. First, most child and adolescent studies have relied on community samples with few studies performed among clinical samples. One exception was a study by Gomez et al. (2019) where clinician-derived data from more than 2,000 Australian children and adolescents were analysed. Consistent with findings from community samples, the data were best explained by two broad dimensions of Internalizing and Externalizing, along with the *p* factor. Whether or not the structure of psychopathology is equivalent in community and clinical samples remains an open question. A study with adults compared a community sample to a clinical sample and found evidence of comparable hierarchical dimensional structures in which Fear (i.e., panic, anxiety) was prominent in an overarching general psychopathology factor, and posttraumatic stress disorder (PTSD) symptoms spanned three dimensions (i.e., Fear, Thought Disorder, and Distress/Disinhibited Negative Affect) (Forbes et al., 2021). On the other hand, Watts et al. (2021) analysed data from three nationally representative samples of adults from the United States and found evidence of *p* in the full samples but no evidence of *p* when the samples were restricted to only those who met criteria for a mental health disorder. Clearly more research with clinical samples, including child and adolescent clinical samples, is required to better understand the structure of psychopathology in this subset of the population.

Second, most studies with children and adolescents have assessed ‘common’ indicators of psychopathology that broadly reflect Internalizing and Externalizing distress, with only a small number of studies having assessed other forms of psychopathology such as psychosis (see Afzali et al., 2018; Carragher et al., 2016 for exceptions) or posttraumatic stress reactions (see Forbes et al., 2021; He & Li, 2021 for exceptions). Moreover, fewer studies have included measures of psychopathology that are especially relevant for children and adolescents such as attention-deficit/hyperactivity (see Michelini et al., 2019 for an exception), separation anxiety (see He & Li, 2021; Vine et al., 2020 for exceptions), emotion dysregulation (see Vine et al., 2020 for an exception), and developmental trauma disorder (DTD; Ford et al., 2018). HiTOP is proposed as an evolving model of psychopathology (Conway et al., 2019), and questions remain about where these types of experiences that are pertinent for children and adolescents are best situated within the model.

The dimensions of psychopathology described in HiTOP are known to be under substantial genetic influence during childhood and adolescence (Allegrini et al., 2020; Waldman et al., 2016). Nonetheless, many other correlates of the dimensions of psychopathology have been identified in child and adolescent samples including biological sex (e.g., women have higher risk of internalizing distress whereas men have higher risk of externalizing distress), reduced global executive functioning, economic deprivation, peer-rejection, negative thinking, and impulsivity (Carragher et al., 2016; Martel et al., 2017; Patalay et al., 2015). A prominent risk factor for all forms of psychopathology is childhood trauma (Alisic et al., 2014; Carliner et al., 2016; McLaughlin et al., 2012). In particular, interpersonal forms of trauma during early life are associated with the highest risk of psychopathology. More research is now required to understand how specific forms of interpersonal trauma are related to the specific dimensions of psychopathology in children and adolescents.

The HiTOP paradigm seeks to empirically discern the structure underlying both symptoms (such as those of PTSD) and personality functions. Therefore, to understand the impact that trauma exposure has on children and adolescents, it is important to assess not only symptoms but also the childhood precursors of personality dysfunctions. Unlike PTSD, DTD was designed specifically to identify trauma-related dysfunctions in the core affective, somatic, cognitive, behavioural, relational, and self functions that are still in flux developmentally in childhood but ultimately can crystallize as personality dysfunction in adulthood (Ford et al., 2018). Given the evidence that attachment trauma may constitute the events that are antecedents for either PTSD or the core self-dysfunction assessed by DTD (Spinazzola et al., 2018, 2021), its association with indicators of both of these trauma-related conditions and of other psychopathology was our focus.

In this study, we used data from a clinical sample of children and adolescents from the United States to address two major study objectives. Our first objective was to determine if multidimensional and hierarchical models consistent with HiTOP could accurately describe our sample data. This objective was approached in a somewhat exploratory manner given the fact that our assessment included phenomena such as developmental trauma disorder (DTD) that are not explicitly recognised in published models of HiTOP (Conway et al., 2019; Kotov et al., 2017). Second, we examined unique associations between specific forms of interpersonal trauma and the specific dimensions of psychopathology. Finally, and based on evidence that dimensions of psychopathology are better predictors of suicidality/self-harm than specific diagnoses, especially the ‘Distress’ subfactor (Conway et al., 2019; Eaton et al., 2013), we assessed how the specific dimensions of psychopathology were uniquely associated with suicidality/self-harm.

## Methods

### Participants

This study is based on a convenience sample of families of 507 children and adolescents aged 7 to 18 years (*M* = 12.11, *SD* = 2.92). The sample included 246 (49%) female participants and 261 (51%) male participants, all of whom were recruited from nine sites in six geographical regions in the United States (Northeast, Mid-Atlantic, South, Midwest, West Coast, Alaska) by referral from mental health, social, work, and paediatric clinicians, and agencies. Participants’ ethnic/racial backgrounds were as follows: 256 (51%) White, 101 (20%) Black or African American, 65 (13%) Hispanic, 11 (2%) Asian, 49 (10%) Biracial, and 13 (3%) reported their race as another race that was unspecified.

## Measures

### Traumatic Experiences Screening Instrument (TESI)

This semi-structured interview assesses eight types of non-interpersonal trauma (accident, illness, death/loss) and 13 types of interpersonal victimization trauma (witness or direct exposure to violence or maltreatment). TESI items have shown evidence of test–retest reliability over a 2–4 month period (Kappa [*K*] = 0.50-0.70) and criterion and predictive validity in psychiatric and paediatric samples (Daviss et al., 2000; Daviss, et al., 2000; Daviss, et al., 2000; Daviss, et al., 2000). Binary variables were calculated for the child’s lifetime history of trauma exposure to represent any occurrence of (1) Non-interpersonal trauma (i.e., accident, illness, or disaster), (2) Traumatic loss, (3) Physical abuse/assault trauma, (4) Witnessing traumatic family violence, (5) Sexual trauma, (6) Witnessing traumatic community violence, (7) Traumatic separation from primary caregiver, (8) Traumatic impairment of primary caregiver, (9) Traumatic Emotional Abuse, and (10) Traumatic neglect (see Table 2). Inter-rater agreement for a random sub-sample of interviews on TESI composite scores was 88–100% (*M* = 97% agreement for both child and parent/guardian interviews).

### Kiddie Schedule for Affective Disorders and Schizophrenia, Present/ Lifetime Version (KSADS/PL)

This semi-structured interview assesses *DSM-IV* child psychiatric disorders with child and parent versions (Kaufman et al., 1997). The screening modules for child psychiatric disorders other than PTSD included gateway symptoms that must be present for a positive diagnosis for each disorder, scored as present = 1 (“threshold”) versus absent = 0 (“not present” or “subthreshold”) (Table 1). Inter-rater agreement across raters for KSADS symptoms of disorders other than PTSD was 78–98% (*M* = 88% and 89% agreement for child and parent/guardian interviews, respectively). PTSD symptoms were assessed with the KSADS module that included all 17 *DSM-IV* symptoms (Present [“threshold”] = 1, Absent = 0 [“subthreshold” or “not present”) in three symptom clusters: re-experiencing (5 items), avoidance (7-items), and arousal (5 items). PTSD symptom questions were asked only if at least one potentially traumatic event was identified and were based on a recall period of the past 30 days. Inter-rater agreement for a random sub-sample of interviews on K-SADS PTSD items was 81–100% (M = 85% and 89% agreement for child and parent/guardian interviews, respectively). For the current analyses, six PTSD items were selected based on research demonstrating their predictive validity for identifying children diagnosed with PTSD (Lang & Connell, 2018): physiological distress when reminded of trauma experiences (B5), avoidance of people, places, or activities that are reminders of trauma experiences (C2), interpersonal detachment (C5), emotional numbing (C6), difficulty sleeping (D1), and difficulty concentrating (D3).

Suicidal Acts, Suicide ideation, and non-suicidal self-injury (NSSI) were assessed with single KSADS screening items, scored as present = 1 (“threshold”) versus absent = 0 (“not present” or “subthreshold”). A suicidal act was defined as a behaviour with a definite suicidal intent. Suicide ideation was defined as often thinking of suicide by a specific method. NSSI was defined as self-harm that is frequent (four or more times a year) or has caused serious injury to oneself (e.g., burn with scarring; broken bone).

### Developmental Trauma Disorder Semi-Structured Interview (DTD-SI)

DTD-SI items were initially designed by experts from the National Child Traumatic Stress Network. After iterative review/revisions, DTD-SI version 10.0 was used in the first phase of this study with *N* = 236 participants (Ford et al., 2018) and version 10.6 was used in the second phase with *N* = 271 participants. The DTD symptoms were identical in both versions of the DTD-SI. Version 10.0 allowed for both threshold and sub-threshold ratings, with either score counted as the symptom being present (Ford et al., 2018). Version 10.6 scored DTD symptoms only as present or absent, based on the symptom occurring with either clinically significant distress or detachment (Ford et al., in press). The 15 DTD symptoms were scored present = 1, absent = 0, organized in three DTD criterion sets: B (4 emotion/somatic dysregulation symptoms), C (five attentional or behavioral dysregulation symptoms), and D (six interpersonal or self- dysregulation symptoms). Each symptom was assessed with a descriptive statement followed by optional probe questions. Inter-rater agreement across raters for a random sub-sample of interviews across all DTD-SI items was 87–100% (*M* = 93.0% agreement on child interviews: 93.5% agreement on parent/guardian interviews). For the current study, only six DTD-SI items were included to match the number of PTSD symptoms. The DTD items also were selected to match the *ICD-*11 complex PTSD symptoms of Disturbances of Self Organization (Shevlin et al., 2018): B1 –emotion dysregulation, B2 –somatic expression of emotion dysregulation; C1 –attention bias toward or away from interpersonal threats; C2 –reckless or conflict provoking behaviour; D1 –self perception as permanently damaged; D2 –attachment insecurity or disorganization.

## Procedure

Interviewers (*N* = 25) viewed simulated demonstration interviews conducted by expert assessors, then independently rated videotaped interviews until achieving > 80% agreement on trauma history, symptoms, and suicidality/NSSI variables with expert ratings. Interviewers conducted and rated videotaped role-play interviews with > 90% agreement with an independent expert reviewer. Interviewers’ first two study interview tapes were reviewed by an independent expert with > 80% agreement on the primary interview variables required. Approximately every fifth interview was randomly selected for independent re-rating (i.e., 73 interviews with a parent or guardian alone, and 36 with the child alone or a parent–child dyad). Interviews were conducted with 245 parent–child dyads, 238 parents alone, and alone with 24 adolescents. Symptoms were considered to be present and traumatic events were considered to have occurred if endorsed by either the parent or child (or both). The study was approved by the University of Connecticut Health Center Institutional Review Board (IE-11–096-2). A parent or legal guardian for each child provided written informed consent, and each child participant provided oral (children under 10 years old) or written (children 10 years and older) assent according to the IRB-approved study protocol.

### Data Analysis

First, descriptive statistics were used to determine endorsement rates of the 45 symptom indicators, the proportion of the sample exposed to each traumatic event, and the proportion of the sample who reported experiencing suicidal ideation, NSSI, and suicidal acts.

Second, the latent structure of the 45 indicators of psychopathology was assessed using confirmatory factor analysis (CFA). Several models consistent with HiTOP were tested. Model 1 was intended to reflect the ‘subfactor’ dimensions of HiTOP and included four factors. ‘Distress’ was measured using 19 items representing experiences of depression, generalized anxiety, posttraumatic stress, and developmental trauma. ‘Fears’ was measured using 12 items representing experiences of phobias, panic attacks, separation anxiety, and obsessive compulsiveness. ‘Externalizing’ was measured using 12 items representing experiences of attention deficit and hyperactivity, conduct disorder, and oppositional defiance. ‘Thought Disorder’ was measured using two items representing psychosis. Model 2 was intended to reflect the ‘spectra’ dimensions of HiTOP and included three factors (Internalizing, Externalizing, and Thought Disorder). The only difference from Model 1 was that the 31 items used to represent the ‘Distress’ and ‘Fears’ were used to represent Internalizing. Model 3 was a hierarchical version of Model 1 where the four factors loaded on to a second-order latent variable reflecting ‘*p*’. A hierarchical version of Model 2 was not tested because such a model is statistically indistinguishable from a first-order factor model (i.e., three factor correlations are replaced by three second-order factor loadings). An exploratory approach was planned that included the inspection of parameters from each model (e.g., patterns of factor loadings and factor correlations) to determine sources of mis- or non-optimal fit, however, decisions to modify models were made primarily on theoretical rather than statistical grounds.

Finally, following the selection of the optimal fitting model, the dimensions of psychopathology were used within a structural equation model (SEM) to identify their unique associations with ten forms of trauma and suicidality/self-harm. The trauma variables were added as observed variables and suicidality/self-harm was modelled as a latent variable measured by the items reflecting suicidal ideation, NSSI, and suicidal acts. Age, sex (0 = male participants, 1 = female participants), and racial identity (0 = Caucasian, 1 = Non-Caucasian) were included as covariates in the model. The SEM model was specified to allow the dimensions of psychopathology to correlate.

All CFA and SEM models were estimated using the mean- and variance-adjusted weighted least squares (WLSMV) estimator as this is appropriate for models with categorical observed variables (Flora & Curran, 2004). Standard recommendations for evaluating model fit were followed (Hu & Bentler, 1999). Acceptable model fit was indicated by a non-significant chi-square (*χ*^*2*^) test result, however, models with significant *χ*^*2*^values should not be rejected given the increased probability of Type 1 errors associated with this test (Tanaka, 1987). Comparative Fit Index (CFI) and Tucker Lewis Index (TLI) values closer to 1 reflect better fit to the sample data, and by convention values greater than 0.90 are typically recommended. Root Mean Square Error of Approximation (RMSEA) and Standardized Root Mean Square Residual (SRMR) values closer to zero reflect better fit to the data, and by convention values less than 0.08 are typically recommended. All analyses were performed in Mplus version 8.2 (Muthén & Muthén, 2013).

## Results

### Descriptive Statistics

Descriptive statistics are presented in Table 1. Endorsement rates for the indicators of psychopathology ranged from 5% (‘psychotic delusions’) to 66% (DTDb1: somatic expression of emotion dysregulation). The most common traumatic event was ‘any non-interpersonal event’ (75%) and the least common was ‘witnessing community violence’ (18%). Suicidal ideation was reported by 18% of the sample, NSSI by 8%, and suicidal acts by 4%.

### CFA Results

CFA model fit results are presented in Table 2. Models 1, 2, and 3 terminated normally however all three were poor representations of the sample data. The RMSEA results suggested reasonable fitting models, however, the CFI, TLI, and SRMR all indicated poor fitting models. Inspection of the parameters (i.e., factor loadings and factor correlations) of each model did not reveal any obvious signs of model misspecification. Moreover, examination of the modification indices did not reveal any signs of serious misspecification. The item measuring ‘irritability and anger’ (intended as a measure of depression) did show evidence of cross-loadings on the ‘Externalizing’ and ‘Thought Disorder’ factors, and although the addition of these cross-factor loadings could be supported on theoretical grounds, the modification index values were such that the addition of these paths was unlikely to substantially affect overall model fit.

Several exploratory based models were then tested, and the model fit results are presented in Table 2. In Model 4 the posttraumatic stress and DTD items were moved from the Distress factor to the Fears factor under the assumption that trauma-related distress may have more in common with anxiety- and fear-based experiences than mood-based experiences. This model, however, was a poor fit to the data. Next, we turned our attention to the separation anxiety items. In Model 5 we retained the posttraumatic stress and DTD items as indicators of Distress and moved the separation anxiety items from the Fears factor to the Distress factor. However, this model also produced poor fit to the sample data. Next, we considered the obsessive–compulsive items. HiTOP includes OCD as part of the ‘Internalizing’ dimension, and specifically as part of the Fear subfactor (Kotov et al., 2017), some studies have modelled these items as part of the ‘Thought Disorder’ dimension (Caspi et al., 2014). Thus, in Model 6, we tested a model with the two obsessive–compulsive items loading on to the Thought Disorder factor rather than the Fear factor. However, this change had little effect on overall model fit.

We then considered the items reflecting trauma-related distress. Prior studies with community samples of adults indicated that posttraumatic stress symptoms may not fit neatly into an Internalizing dimension (Forbes et al., 2021; Hyland et al., 2021). Thus, we assessed if the posttraumatic stress and DTD symptoms represented a distinct dimension of psychopathology. In Model 7 we included five factors of Distress, Fear, Externalizing, Thought Disorder, and Traumatic Stress. This change led to a substantial improvement in overall model fit with the RMSEA result indicating very close fit to the sample data, and the CFI and TLI results indicating acceptable fit to the sample data. Although the SRMR remained outside the recommended range for acceptable model fit, this model was deemed to be an acceptable representation of the latent structure of psychopathology in this sample.

With an acceptable fitting model, we then tested if a hierarchical version of this model (i.e., the addition of a second order *p* factor) also provided acceptable fit. This model (Model 8) yielded similar fit to the correlated first-order factor model. The standardized factor loading for Distress on *p* was greater than 1.0 which, while technically outside the bounds of normal standardized estimates, does not necessarily indicate a mis-specified model (Deegan, 1978; Jöreskog, 1999). Models 7 and 8 were, therefore, both deemed to be plausible representations of the sample data, and the parameter estimates for these models are presented in Table 3.

### SEM Results

We specified a SEM model with 13 exogenous variables (ten forms of trauma, age, sex, and racial identity) predicting five correlated dimensions of psychopathology (Distress, Fears, Externalizing, Thought Disorder, and Traumatic Stress) which subsequently predicted a latent variable of suicidality/self-harm. This model fit the sample data reasonably well (*χ*^*2*^ (1624) = 2510, *p* < 0.001; CFI = 0.90; TLI = 0.90; RMSEA = 0.03 [90% CI = 0.03, 0.04], SRMR = 0.11), and explained 17% of variance in Distress (*p* < 0.001), 9% of variance in Fear (*p* = 0.001), 6% of variance in Traumatic Stress (*p* = 0.010), 8% of variance in Externalizing (*p* = 0.002), 22% of variance in Thought Disorder (*p* = 0.001), and 37% of variance in suicidality/self-harm (*p* < 0.001). The full set of standardized regression coefficients are presented in Tables 4 and 5.

Distress was significantly associated with caregiver separation, caregiver impairment, physical abuse, and sexual abuse. Fear was significantly associated with traumatic loss. Traumatic Stress was significantly associated with witnessing family violence and older age. Externalizing was significantly associated with caregiver impairment and physical abuse. Thought Disorder was significantly associated with sexual abuse and physical abuse. Additionally, Distress was significantly and positively associated with suicidality/self-harm (β = 0.67, *p* < 0.001), while Fear was significantly and negatively associated with suicidality/self-harm (β = -0.24, *p* = 0.046).

We then respecified the model replacing the five dimensions of psychopathology with the higher-order *p* factor. This model was also a reasonable approximation of the sample data (*χ*^*2*^ (1685) = 2548, *p* < 0.001; CFI = 0.91; TLI = 0.90; RMSEA = 0.03 [90% CI = 0.03, 0.04], SRMR = 0.11), and explained 18% of variance in *p* (*p* < 0.001) and 32% of variance in suicidality/self-harm (*p* < 0.001). The standardized regression coefficients are also reported in Tables 4 and 5. *p* was significantly associated with caregiver impairment, physical abuse, and sexual abuse. Moreover, *p* was significantly and positively associated with suicidality (β = 0.56, *p* < 0.001).

## Discussion

In this study, we examined the latent structure of psychopathology in a clinical sample of children and adolescents, and how different dimensions of psychopathology were uniquely associated with multiple traumatic events, as well as with suicidality/self-harm. We found that the latent structure of psychopathology could be reasonably represented by five latent factors representing Fear, Distress, Externalizing, Thought Disorder, and Traumatic Stress, and that the correlations between these factors could be explained by a higher-order general factor of psychopathology (i.e., *p*). It is important to stress that considerable exploration of our sample data was required before an adequate fitting model could be found, therefore caution is warranted in the interpretation of these findings. Although the optimal fitting model(s) in this sample was consistent with the HiTOP framework, the most notable deviation from HiTOP was the need to include a distinct factor representing Traumatic Stress psychopathology. This finding adds to a small-but-growing literature suggesting that trauma-related symptoms may not be optimally located within the broad Internalizing domain and suggests that it may warrant its own factor (Forbes et al., 2021; Hyland et al., 2021). However, given the novelty of this finding, we call for considerably more research to be performed before drawing any conclusions about the substantive nature of, or need for, a Traumatic Stress factor within an overall model of child and adolescent (or, indeed, adult) psychopathology.

It was noteworthy that the correlations among the Distress, Fear, Externalizing, and Thought Disorder factors were all moderate or large while their associations with the Traumatic Stress factor were either weak (with Distress and Externalizing) or non-significant (with Fear and Thought Disorder). This could be interpreted in two ways. One is that Traumatic Stress symptomatology is reasonably independent of other forms of psychopathology. This is plausible given the requirement of traumatic exposure for these symptoms. The other is that the Traumatic Stress factor is a statistical/methodological artefact. It is possible that this factor emerged from the common method variance shared across the indicators of traumatic stress. As such, future studies should include a wider array of indicators of traumatic stress symptomatology that are more orthogonal in their design. Only future research will reveal which is more likely, but we believe these findings provide an empirical basis to investigate this issue.

Several unique associations were identified between the dimensions of psychopathology and the different forms of traumatic exposure. Traumatic caregiver impairment, physical abuse, and sexual abuse were associated with multiple dimensions of psychopathology, including the *p* factor. Traumatic caregiver separation, traumatic loss, and family violence were associated with the Distress, Fear, and Traumatic Stress factors, respectively. Previous research has found that traumatic separation from a caregiver was the only unique trauma predictor of recurrent depression in adults (Gloger et al., 2021). Additionally, previous research has found that traumatic caregiver separation was a significant predictor of DTD symptomatology among children and adolescents (Spinazzola et al., 2021). The lack of association between traumatic caregiver separation and the Traumatic Stress factor in this study may be due to the inclusion of the additional dependent variables (i.e., the other dimensions of psychopathology). In other words, the previously identified association between traumatic caregiver separation and DTD symptoms may have been due to comorbidity between Distress- and Traumatic Stress-based disorders and symptoms, as observed in previous research with children and adolescents (van der Kolk et al., 2019). The association between traumatic loss and Fear symptomatology is in line with previous research showing that negative life events, particularly events relating to death, play a significant role in the onset of multiple Fear-based disorders such as generalised anxiety disorder and panic disorder (Schiele & Domschke, 2018). The finding that the primary trauma experience correlated with of trauma-related symptoms was family violence is consistent with the DTD formulation of traumatic victimization and attachment disruption (Spinazzola et al., 2018, 2021).

We found a strong positive association between *p* and suicidality/self-harm, and when suicidality/self-harm was correlated with the different dimensions of psychopathology, an interesting pattern of associations emerged. The Distress factor was positively associated with suicidality/self-harm while the Fear factor was negatively associated with suicidality/self-harm. Furthermore, the Traumatic Stress, Externalizing, and Though Disorder dimensions were not associated with suicidality/self-harm. These findings are in-line with previous findings among adults showing that the Distress factor is particularly strongly related to suicide-related variables (Conway et al., 2019; Eaton et al., 2013). Furthermore, although Distress and Fear both reflect Internalizing psychopathology, the discrepant associations with suicidality/self-harm supports the distinction between these subfactors in the HiTOP model. These findings suggests that Distress symptomatology may explain previously observed relationship between suicidality and other dimensions of psychopathology that have been observed throughout the literature (e.g., Chapman et al., 2015; DeVylder et al., 2015; Hyland, Rochford, et al., 2021; Lüdtke et al., 2018; Pickles et al., 2010; Smith et al., 2018; Zahid & Upthegrove, 2017).

These findings have several research and clinical implications. First, HiTOP is conceived as evolving model (Conway et al., 2019) and we have provided evidence that there may be a distinct dimension of psychopathology related to trauma reactions in children and adolescents. Future research will be needed to determine if this observation replicates in other child/adolescent samples, and in adult samples, but current findings open a potentially interesting line of research for how to advance to the HiTOP model. Second, we found evidence of unique associations between specific forms of trauma and specific dimensions of psychopathology. These findings add to a growing understanding of the traumatic antecedents of different dimensions of psychopathology. Future research may benefit from exploring how other types of childhood adversities and traumas (such as those events represented in the ACE literature) relate to different dimensions of psychopathology. Moreover, these findings may be helpful for clinicians in determining what symptoms are more or less likely to occur depending upon a patient’s trauma history. Third, given the unique association between Distress and suicidality/self-harm, clinicians working with patients presenting a broad array of these symptoms should be acutely aware of the risk of suicide/self-harm.

The study had several limitations worth noting. First, the reliance on parental reports for most participating children is a limitation given the established discrepancies between parental and child reports (e.g., Korelitz & Garber, 2016). Second, the analytic sample was constructed by recruiting children and adolescents recruited from multiple, diverse sites. Given the limited sample size across these sites, it was not possible to examine the measurement invariance across sites. As such, we assumed measurement invariance across sites and results should be interpreted with caution. Third, the sample of children and adolescents resided in the United States therefore these findings may not be generalisable to other nations. Second, although we measured many psychiatric symptoms across multiple dimensions of psychopathology, some dimensions were represented by a small number of symptoms (e.g., Thought Disorder) and others were not represented at all (e.g., Mania, Eating Pathology). Third, the cross-sectional design means that no inferences can be made regarding the temporal relationships between trauma exposure and psychopathology, and between psychopathology and suicidality. Fourth, parental education, income, and overall socioeconomic status was not assessed in the current study but should be included in future research in light of its documented association with children’s psychiatric symptoms.

In conclusion, our findings indicate that the latent structure of psychopathology in this clinical sample of children and adolescents can be effectively described in terms of a multidimensional and hierarchical model. While generally consistent with the HiTOP framework, our findings suggest that a distinct dimension of Traumatic Stress might exist for children and adolescents. Much more research is needed before any revision to the HiTOP model should be considered but it does raise the possibility of a modification to the model. In addition, we demonstrated unique associations between multiple forms of trauma exposure and different dimensions of psychopathology, and this can help to elucidate different developmental pathways to different expressions of psychopathology. Finally, we also found evidence that Distress-related psychopathology is particularly relevant to suicidality/self-harm in children and adolescents.

**Table 1:** Endorsement rates for each symptom, indicator of suicidality, and trauma exposure

	Mental health problem	Scale	Endorsement %
1	Depressed mood	Dep1	33
2	Irritability and anger	Dep2	39
3	Anhedonia, lack of interest, low motivation, boredom	Dep3	24
4	Overanxious, unrealistic worry about future	GAD1	22
5	Somatic complaints	GAD2	22
6	Marked self-consciousness	GAD3	27
7	Marked feeling of tension/unable to relax	GAD4	27
8	Somatic distress due to trauma reminders	PTSD1	32
9	Avoidance of people, places, activities	PTSD2	36
10	Interpersonal detachment	PTSD3	36
11	Emotional numbing	PTSD4	25
12	Sleep problems	PTSD5	39
13	Concentration problems	PTSD6	44
14	Emotion dysregulation	DTD1	66
15	Somatic expression of emotion dysregulation	DTD2	33
16	Attention bias toward or away from threats	DTD3	41
17	Reckless or conflict-provoking behaviour	DTD4	9
18	Self-perception as permanently damaged	DTD5	26
19	Attachment insecurity or disorganization	DTD6	28
20	Avoidant disorder/social phobia-shrinks from contact	Phobia1	12
21	Fear of social situations	Phobia2	16
22	Agoraphobia and specific phobias-distress	Phobia3	17
23	Avoidance	Phobia4	18
24	Panic attacks	PA	8
25	Fears calamitous event that will cause separation	SAD1	18
26	Fears harm befalling attachment figure	SAD2	22
27	School reluctance refusal	SAD3	10
28	Fears sleeping away from home/sleeping along	SAD4	15
29	Fears being alone at home	SAD5	18
30	Compulsions	OCD1	8
31	Obsessions	OCD2	8
32	Difficulty sustaining attention on tasks or play activities	ADHD1	42
33	Easily distracted	ADHD2	45
34	Difficulty remaining seated	ADHD3	30
35	Impulsivity	ADHD4	38
36	Lies	CP1	12
37	Truant	CP2	6
38	Initiates physical fights	CP3	7
39	Bullies, threatens, or intimidates others	CP4	11
40	Non-aggressive stealing	CP5	10
41	Loses temper	ODD1	38
42	Argues a lot with adults	ODD2	33
43	Disobeys rules a lot	ODD3	26
44	Hallucinations	Psy1	8
45	Delusions	Psy2	5
***Suicidality***			
1	Suicidal ideation		18
2	Non-suicidal physical self-damaging acts		8
3	Suicidal acts		4
***Traumatic life events***			
1	Non-interpersonal trauma		75
2	Traumatic loss		49
3	Traumatic caregiver separation		54
4	Traumatic caregiver impairment		42
5	Physical abuse		53
6	Sexual abuse		21
7	Family violence		39
8	Community violence		18
9	Emotional abuse		20
10	Neglect		19

*Dep* Depression, *GAD* Generalized Anxiety Disorder, *PTSD* Posttraumatic Stress Disorder, *DTD* Developmental Trauma Disorder, *PA* Panic Attack, *SAD* Separation Anxiety Disorder, *OCD* Obsessive Compulsive Disorder, *ADHD* Attention Deficit Hyperactivity Disorder, *CP* Conduct Problems, *ODD* Oppositional Defiance Disorder, *Psy* Psychosis

**Table 2:** Model fit results for the alternative models of the latent structure of psychopathology

	*χ*^*2*^	df	CFI	TLI	RMSEA (90% CI)	SRMR
***Confirmatory models***						
Model 1: ‘Subfactor’ model (D, F, E, TD)	2915*	939	0.79	0.77	0.06 (0.06, 0.07)	0.17
Model 2: ‘Spectra’ model (I, E, TD)	3402*	942	0.73	0.72	0.07 (0.07, 0.07)	0.17
Model 3: Hierarchical ‘subfactor’ model (D, F, E, TD, *p*)	2928*	941	0.79	0.77	0.07 (0.06, 0.07)	0.17
***Exploratory models***						
Model 4: Modified ‘subfactor’ model; trauma-related items on Fear	2995*	939	0.78	0.77	0.07 (0.06, 0.07)	0.17
Model 5: Modified ‘subfactor’ model; separation anxiety items on Distress	3045*	939	0.77	0.76	0.07 (0.06, 0.07)	0.17
Model 6: Modified ‘subfactor’ model; obsessive–compulsive items on TD	2878*	939	0.79	0.78	0.06 (0.06, 0.07)	0.17
Model 7: Modified ‘subfactor’ model; distinct Traumatic Stress factor	1619*	935	0.93	0.92	0.04 (0.04, 0.04)	0.12
Model 8: Modified ‘subfactor’ model Traumatic Stress factor with ‘*p*’	1624*	940	0.93	0.92	0.04 (0.04, 0.04)	0.12

*N* 507, *D* Distress, *F* Fear, *TD *Thought Disorder, *I* Internalizing, *E* Externalizing, *p* General Psychopathology, *χ*^*2*^ chi-square test, df degrees of freedom, *CFI* Comparative Fit Index, *TLI* Tucker Lewis Index, *RMSEA* (90% CI) Root Mean Square Error of Approximation with 90% confidence intervals, *SRMR* Standardized Root Mean Square Residual

^*^ Indicates *χ*^*2*^ is statistically significant (*p* < 0.001)

**Table 3:** Factor loadings and factor correlations from the best-fitting dimensional models of psychopathology

	Distress	Traumatic Stress	Fear	Externalizing	Thought Disorder
***First-order factor loadings***					
Depressed mood	0.64				
Irritability and anger	0.80				
Anhedonia, low motivation	0.68				
Overanxious/unrealistic worry	0.64				
Somatic complaints	0.60				
Marked self-consciousness	0.65				
Marked feeling of tension	0.80				
Somatic distress due to trauma		0.76			
Avoidance of trauma reminders		0.74			
Interpersonal detachment		0.83			
Emotional numbing		0.68			
Sleep problems		0.70			
Concentration problems		0.73			
Emotion dysregulation		0.69			
Somatic dysregulation		0.68			
Attention bias		0.70			
Reckless behaviour		0.57			
Self-perception damaged		0.61			
Attachment insecurity		0.61			
Social phobia			0.62		
Fear of social situations			0.67		
Agoraphobia			0.90		
Avoidance			0.91		
Panic attacks			0.52		
Fears about separation			0.78		
Harm to attachment figure			0.77		
School reluctance refusal			0.63		
Fears sleeping away from home			0.65		
Fears being alone at home			0.68		
Compulsions			0.59		
Obsessions			0.74		
Difficulty sustaining attention				0.90	
Easily distracted				0.97	
Difficulty remaining seated				0.86	
Impulsivity				0.84	
Lies				0.62	
Truant				0.40	
Initiates physical fights				0.65	
Bullying/threatening others				0.66	
Non-aggressive stealing				0.60	
Loses temper				0.81	
Argues a lot with adults				0.86	
Disobeys rules a lot				0.80	
hallucinations					0.90
Delusions					0.83
***Second-order factor loadings on p***	1.03	0.14*	0.65	0.62	0.61
***Factor correlations***					
Distress	1				
Traumatic Stress	0.16*	1			
Fear	0.69	0.05^ ns^	1		
Externalizing	0.64	0.13*	0.38	1	
Thought Disorder	0.45	-0.17^ ns^	0.49	0.48	1

All factor loadings and factor correlations are statistically significant (*p* <0.001) except * (*p* < 0.050) and ^ns^ (not significant)

**Table 4:** Standardized regression coefficients of each form of trauma on the dimensions of psychopathology

	**Correlated factor model**					**Higher-order Model**
	Distress	Fears	Traumatic stress	Externalizing	Thought Disorder	*p*
Non-interpersonal trauma	0.04	-0.05	0.01	-0.02	0.10	0.00
Traumatic loss	0.07	0.12*	0.10	0.04	-0.11	0.09
Traumatic caregiver separation	0.11*	0.06	0.07	0.01	-0.15	0.07
Traumatic caregiver impairment	0.20***	0.10	-0.07	0.21***	0.09	0.22***
Physical abuse	0.16**	0.10	0.05	0.12*	0.23*	0.17***
Sexual abuse	0.12*	0.05	0.02	0.08	0.23**	0.12*
Family violence	0.02	0.09	0.11*	-0.02	0.15	0.05
Community violence	0.06	0.05	0.03	0.05	0.05	0.08
Emotional abuse	0.00	-0.03	-0.01	0.02	-0.03	0.00
Neglect	-0.07	0.06	-0.04	-0.07	0.02	-0.05
Age	-0.02	-0.05	0.12**	-0.05	-0.01	-0.04
Sex (Female participants)	0.04	0.06	0.04	-0.01	-0.15	0.03
Racial identity (Non-Caucasian)	0.00	0.00	0.04	0.01	-0.08	0.00
R^2^	0.17***	0.10***	0.06**	0.08**	0.22***	0.18***

Statistical significance = *p < .050, **p < .010, ***p < .001; R^2^ = proportion of variance explained

**Table 5:** Standardized regression coefficients for each dimension of psychopathology on suicidality

	Suicidality
***Correlated factor model***	
Distress	0.67***
Fear	-0.24*
Traumatic Stress	0.07
Externalizing	-0.04
Thought Disorder	0.20
R^2^	0.37***
***Higher-order model***	
*p*	0.56***
R^2^	0.32***

Statistical significance = *p < 0.050, **p < 0.010, ***p < 0.001, R^2^ = proportion of variance explained
